# An improved multi-scale branching convolutional neural network for rolling bearing fault diagnosis

**DOI:** 10.1371/journal.pone.0291353

**Published:** 2023-09-13

**Authors:** Meng Xu, Yaowei Shi, Minqiang Deng, Yang Liu, Xue Ding, Aidong Deng

**Affiliations:** National Engineering Research Center of Power Generation Control and Safety, School of Energy and Environment, Southeast University, Nanjing, China; Hainan Normal University, CHINA

## Abstract

The vibration signals measured in practical engineering are usually complex and noisy, which brings challenges to fault diagnosis. In addition, industrial scenarios also put forward higher requirements for the accuracy and computational efficiency of diagnostic models. Aiming at these problems, an improved multiscale branching convolutional neural network is proposed for rolling bearing fault diagnosis. The proposed method first applies the multiscale feature learning strategy to extract rich and compelling fault information from diverse and complex vibration signals. Further, the lightweight dynamic separable convolution is elaborated and coupled into the feature extractor to "slim down" the model, reduce the computational loss on the one hand, and further improve the model’s adaptive learning ability for different inputs hand. Extensive experiments indicate that the proposed method is significantly improved compared with existing multi-scale neural networks.

## 1. Introduction

Rolling bearing is the joint of rotating machinery, which plays a vital role in industrial production [1, 2]. However, under the complex working conditions of intense, long-term noise, variable speed, and variable load, rolling bearings are prone to wear, fracture, or other failures, which rapidly lead to the collapse of the overall performance of the equipment and even cause irreparable economic losses and severe personnel safety problems [3, 4]. Therefore, it is of great practical significance to fully perceive and identify the running condition of rolling bearings to improve the reliability of rotating machinery system and mitigate the risk of unplanned shutdown.

The diagnostic technology based on vibration signals can be divided into non-machine learning, traditional machine learning methods, and deep learning methods. Non-machine learning methods include time-domain, frequency domain, and time-frequency analysis [5–10]. However, with the expansion of fault data and the complexity of the operating environment, it is difficult to obtain better diagnostic accuracy by relying on the traditional diagnosis method of manually extracting signal fault characteristics.

Fault diagnosis based on machine learning has obtained more research and good results in recent years. However, several obvious drawbacks still exist. For instance, the manually extracted features largely rely on expert experience in specific fields, and it is difficult to thoroughly describe the complex dynamic characteristics of rotating machinery. As an end-to-end approach, deep learning with excellent autonomous feature learning and recognition decision capacity is well suited to the above drawbacks. It has been investigated extensively in the diagnosis of mechanical faults [11, 12] as one of the essential branches of deep learning networks, the convolutional neural network (CNN) has achieved remarkable success in fault diagnosis with its unique feature learning mechanism and powerful classification capability [13–16].

Li et al. [17] proposed the two-dimensional time-frequency spectrogram after a short-time Fourier transforms as the input of CNN, which improves the robustness of the model. Han et al. [18] obtained the corresponding two-dimensional feature maps by wavelet transformation of the original signal and achieved an intelligent compound fault diagnosis of rolling bearings through CNN. Xiong et al. [19] proposed a data preprocessing method based on dimensionless Gram matrix, which achieved satisfactory results in mechanical fault diagnosis. It is remarkable that in fault diagnosis, the vibration signal obtained by the most commonly used acceleration sensor is a one-dimensional (1D) time series, not a two-dimensional (2D) time-series graph. The mentioned works will be converted from 1D signals into 2D feature images and then fed into the model to materialize the evaluation results. However, the inherent vibration information of the device embedded in the 2D feature map is difficult to be accessed by the CNN model. Compared with 1D signals, 2D characteristic images may be lost some helpful information in the 1D-to-2D process. To attack this challenge, Wu et al. suggested a one-dimensional CNN that integrates feature recognition and autonomous diagnosis with the powerful learning capability of generalized fault features for processing raw vibration signals that contain more discriminable information [20]. Zhang et al. established a deep CNN, using the original vibration signal as model input for feature extraction and fault classification identification of rolling bearings. They demonstrated that using the original 1D time-series signal as the input of the CNN model has good generalization ability and diagnostic stability in complex operational environments [21, 22]. Peng et al. integrated residual learning into 1d-cnn network and achieved good results in structural damage location of high-speed rail bearings [23].

Usually, the measured vibration signal contains more sudden changes in harsh working conditions. If there is a fault shock, it is more likely to be submerged in the sudden change in environmental noise [24, 25]. This leads to vast differences in fault information [26, 27]. As a result, the vibration signal contains complex patterns on the time scale. Thus, single-scale CNNs are difficult to be applied to the fault diagnosis task of rolling bearings and have poor performance in them. The multi-scale learning method can capture the features contained in the original signal from multiple scales, and can calmly cope with the above challenges, which has been widely concerned by researchers. It is also demonstrated in [28] that multi-scale feature extraction can better contain more discriminative information in multi-scale time than single-scale feature extraction for signal extraction. In [29], an MS-CNN architecture is proposed. Multiple down-sampling and pooling operations are performed in parallel on the original vibration signal to obtain multi-scale information. Different scale feature information is extracted by convolution-pooling blocks, which shows more promising results than single-scale CNN. In [30], B et al. proposed an MC-CNN network to add a multi-scale information fusion cascade layer to the original CNN network to enhance the ability to extract the time-scale information of the input signal. They verified the effectiveness of the multi-scale cascade layer. Although the previous work makes up for the deficiency of traditional single-scale CNN in feature extraction capability and achieves better diagnostic results, for the strong noise case in industrial applications, the extraction of fault-related shock segments from the vibration signal is insufficient, which prevents the shock segments from receiving more attention. Ding et al. [31] proposed an MSACNN architecture to achieve the extraction of sensitive features in different dimensions by transforming a 1D time series into a 2D image as the input to the model, broadening the width of the network using a dual-scale convolutional structure, and focusing on key features in different dimensions using an attention mechanism. Although the above work introduces the feature attention mechanism and focuses well on the information with differentiation, it fails to verify the effect of more convolutional scales on the model. Xu et al. [32] developed an IMS-FACNN model to obtain multi-timescale information by acquiring the mean data through IMS fine-grained sliding window continuous displacement and introducing a feature focus mechanism after the feature extraction process, which can focus on multi-scale fault identifiable information. The model improves the anti-interference ability and diagnostic performance of the signal. All of the above works have demonstrated that introducing attention mechanisms into multi-scale convolutional network structures can effectively improve the diagnostic performance of the models. In addition, some studies have shown that more branches and wider convolution kernels can improve the recognition accuracy of the model. Still, too many parameters and more complex structures will increase the memory and calculation cost of the model, which does not conform to the requirements of industrial Internet applications. The Industrial Internet of Things requires models to have high accuracy while requiring less storage and computing costs.

To tackle the above problems and meet the needs of practical industrial applications, this paper proposed a stackable multi-scale branching attention feature extraction module (MBAFEM), and an intelligent fault diagnosis network based on MBAFEM is constructed. The method considers the characteristics of different time scales of the original feature information, allowing the faulted shock segment to be noticed in focus on the effective channel. By designing multi-scale branches with lightweight dynamically separable convolutions, deeper fault-related information can be extracted from the original vibration signals at multiple time scales to better represent the classifiable fault characteristics. At the same time, the complexity of the model can be diminished to reduce the number of network parameters of the model. MBAFEM adopts the simple stacking structure design, which overcomes the existing multi-scale CNN structure complexity and drawbacks. Stacking multiple MBAFEMs will lead to a rapid increase in network depth. The parameter gradient blast/vanishing caused by the increasing depth of the model network is minimized by introducing residual connectivity techniques.

1. Drawing on profound separable and dynamic convolution ideas, a lightweight dynamical separable convolution (LDSC) is proposed and introduced into the multi-scale information extraction module, which can effectively capture the failure characteristics of bearings. Inheriting the advantages of dynamic convolution and separable convolution, the proposed dynamic separable convolution can not only learn more dimensional bearing failure characteristics. The experimental results from subsection 4.2 show that it improves the diagnostic performance while minimizing the number of parameters and the size of the model.
2. The effective channel attention mechanism is introduced into MBAFEM after the multi-scale branching feature extraction layer. On the one hand, it focuses on the feature information of different channels by adaptive one-dimensional convolution and assigns weights to different scales. On the other hand, this attention mechanism avoids downscaling and effectively captures cross-channel interactions, compensates for the lack of weight sharing in the convolution kernel, and makes the model pay more attention to the impact features in the signal. To evaluate the diagnostic performance of the proposed method, we constructed a real-world application scenario with solid noise on the CWRU dataset. The comparison with existing work verifies the effectiveness and superiority of the proposed method.

The rest of this paper is summarized below. Section 2 describes the proposed fault diagnosis model based on the MBAFEM module in detail. Section 3 performs the data description and experimental validation setup. Section 4 presents the experimental results obtained using actual bearing data, and the proposed method is evaluated on its excellent performance. Finally, Section 5 is concluded with a summary of the whole paper.

## 2. Proposed approach

As described above, vibration signals generated by rolling bearings under multiple working conditions and intense noise have multi-timescale characteristics and hidden nature. There is a need for more single-scale CNN to extract these fault features. For this reason, some people adopt multi-scale CNN models for fault diagnosis to obtain more accurate results [33–35]. Based on this, a multi-scale branching feature extractor is proposed in this paper that combines LDSC, channel attention mechanism, and residual learning into a multi-scale CNN. As a result, MBAFEM has excellent fault diagnosis accuracy and robust noise immunity.

### 2.1 Lightweight dynamic separable convolution

**Fig 1:**
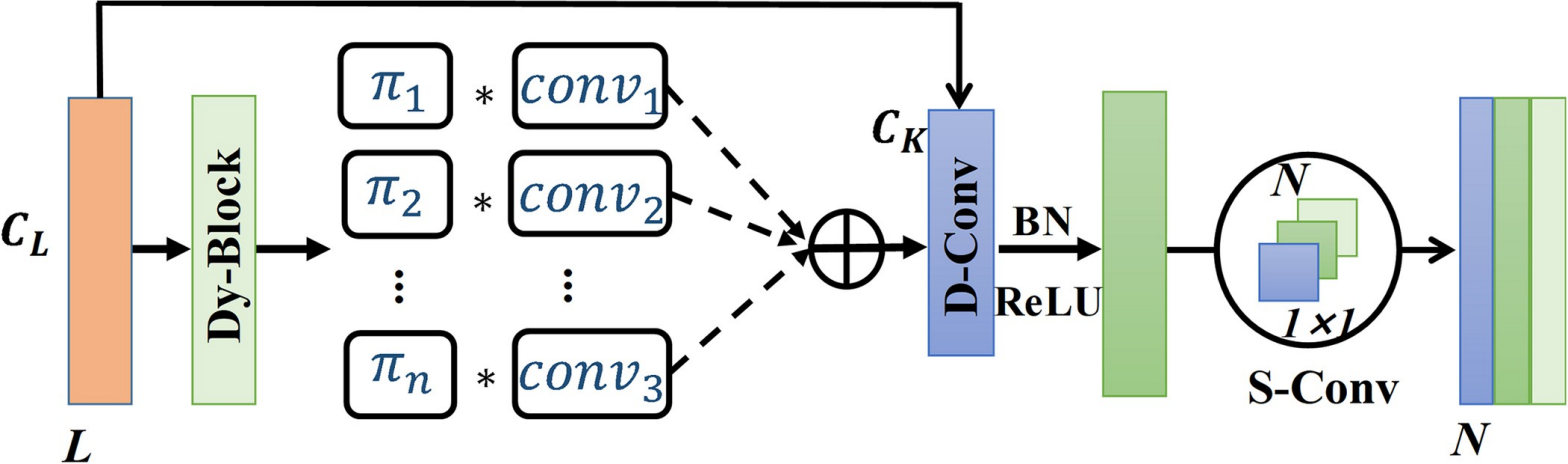
The basic structure of the lightweight dynamic separable convolution.

**Fig 2:**
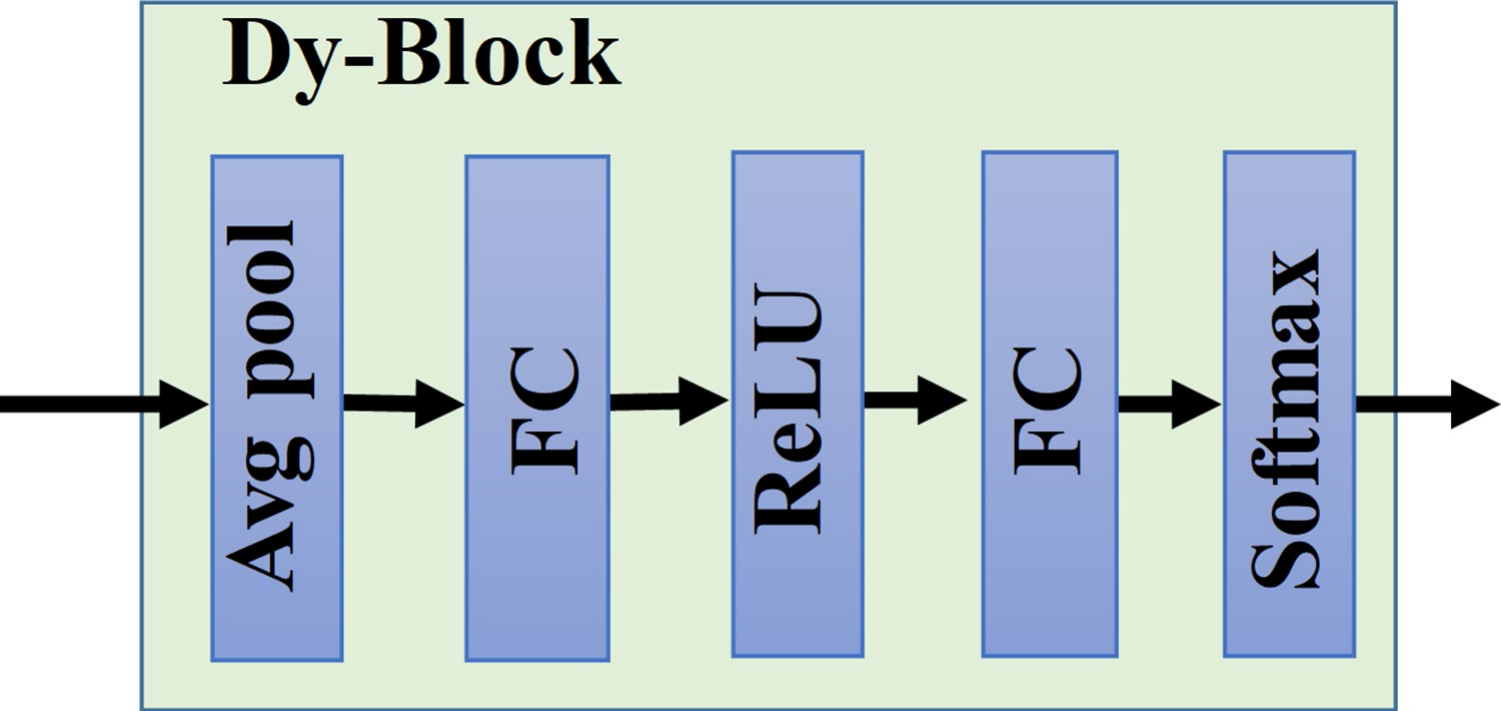
The structure of Dy-Block.

In this work, we propose a lightweight dynamic separable convolution method that combines the advantages of dynamic convolution [36, 37] and depth-separable convolution [38], as shown in Fig 1. The unique feature is that the LDSC improves the expressiveness of the model and reduces the computer storage cost, borrowing the structure of deep separable convolution and using distributed convolution in 2 steps. The first step is dynamic channel convolution, which incorporates the idea of dynamic convolution based on channel convolution, and dynamically aggregates multiple parallel convolution kernels by fusing attention mechanisms to perform one attention operation on the input and then obtains the weight of each convolution kernel, superimposes the learned weights on different convolution kernels to achieve a dynamic selection of convolution kernels, and performs convolution operation after performing dynamic aggregation of multiple convolution kernels. The dynamic branching structure of which is shown in Fig 2. Compared with the traditional static convolution (single convolutional kernel per layer), the attention-based dynamic superposition of multiple convolutional kernels significantly improves expressiveness, and the additional computational cost is negligible. In the second step, the form of point convolution is taken to eliminate the dynamic convolution that makes the number of parameters too large. The dynamic separable convolution has the advantages of both dynamic convolution and depth separable convolution, and the dynamic superposition of multiple convolution kernels on the vector not only improves the model expression capability but also significantly reduces the model parameters, lowers the model storage cost and enhances the applicability of the model for industrial applications. It is worth noting that dynamic separable convolution is used in this paper only in the multi-scale branching feature extractor. We will later verify the superiority of dynamic separable convolution compared with deep separable convolution in subsection 4.2.

### 2.2 Efficient channel attention-based enhanced feature identification mechanism (ECA-EFIM)

The impact segment usually expresses the characteristics that reflect the fault behavior of rolling bearings in vibration signals. To better extract the fault behavior characteristics, we should focus on the shock information and highlight the fault information of rolling bearings to improve feature extraction’s relevance, reliability, and interpretability. Consequently, the Efficient Channel Attention (ECA) mechanism is introduced in the feature extraction module. The ECA module was proposed in 2020, in which channel attention is set to squeeze and stimulate the convolutional learning block and avoid dimensionality reduction, which can effectively capture local cross-channel interaction information [39].

**Fig 3:**
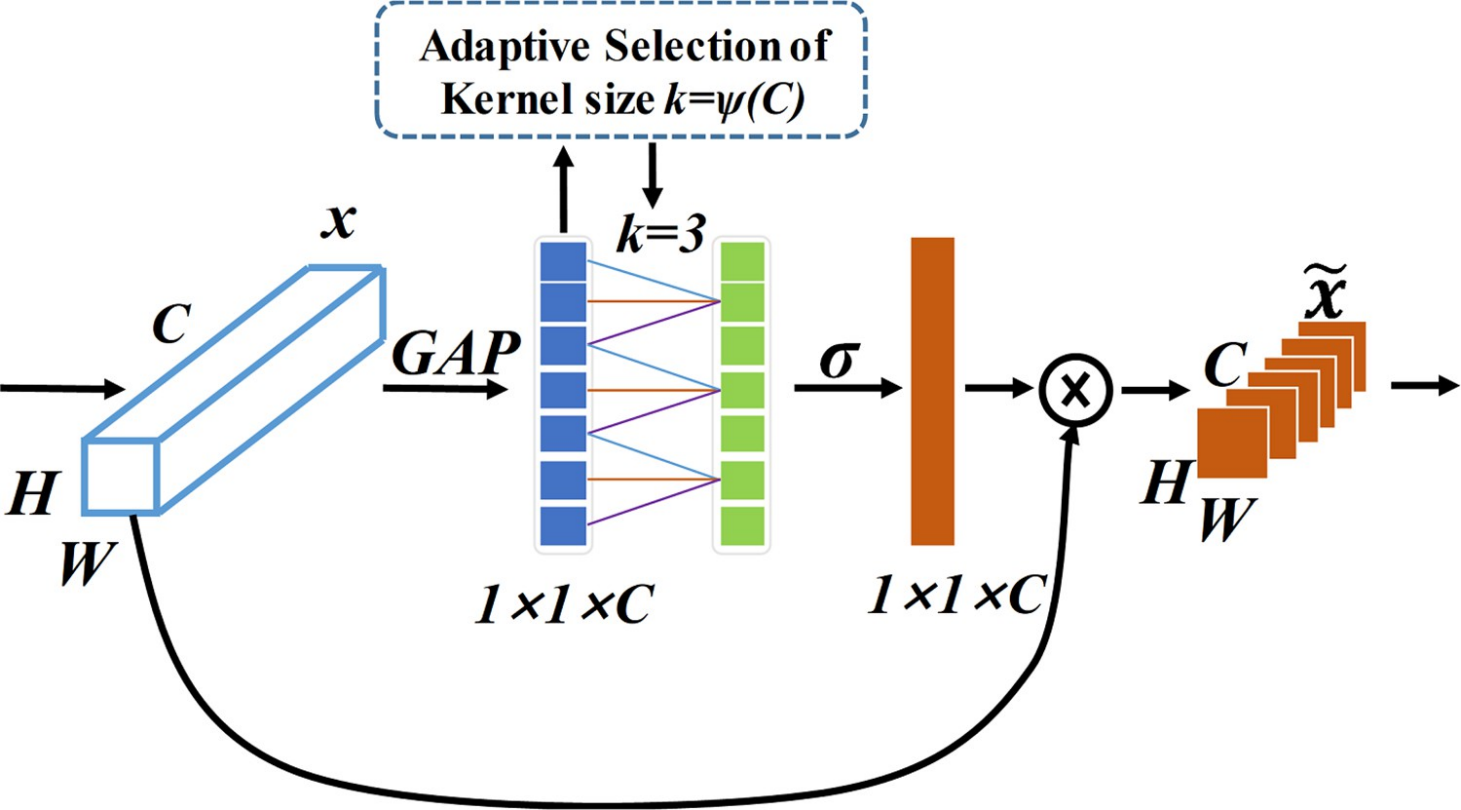
Diagram of ECA.

Fig 3 shows the ECA module, with the original input features on the far left. The ECA module no longer performs the dimensionality reduction operation and directly performs global average pooling (GAP) on the input sequence to obtain all features without dimensionality reduction. Based on this, the ECA efficiently captures all features without dimensionality reduction by adaptively selecting a fast one-dimensional size *k* (1D-convolution) of size *k* to effectively capture the local cross-channel interaction information. Then the feature weight ratio of each channel is generated by sigmoid function. Channel weights are used to combine with original input features to obtain channel features with different levels of attention, so that different weights of all features can be accurately captured and focus on key features can be enhanced. The network constructed with this module is easier to extract the input features based on the channel dimension. Furthermore, it brings significant improvement with little increase in model complexity.

**Fig 4:**
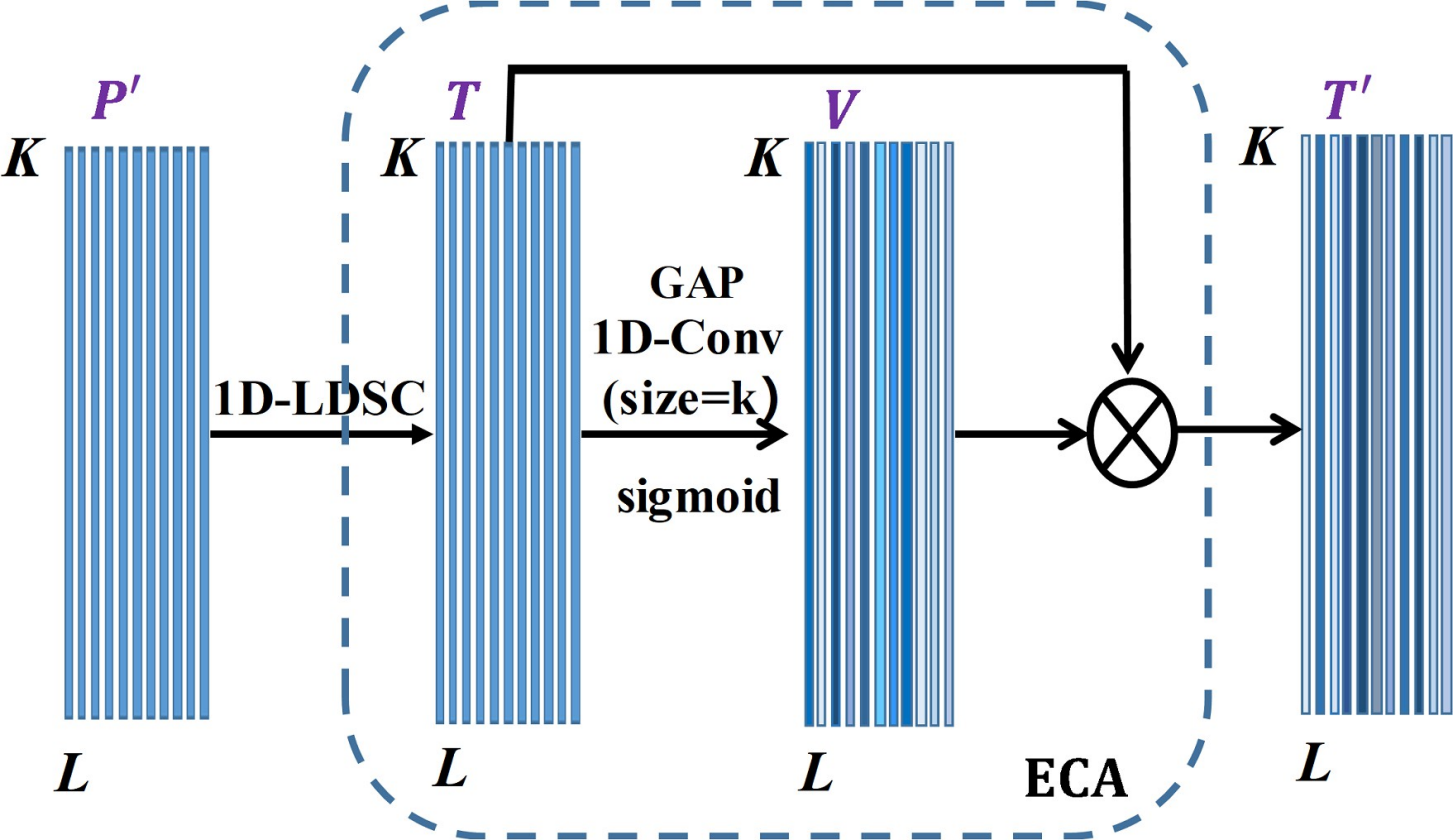
The basic structure of the ECA-EFIM.

The complete structure diagram of ECA-EFIM is drawn in Fig 4. The ECA module and 1D-LDSC are used in EFIM, which is a combined attention feature extraction mechanism. Suppose the input is $P^{\prime }=[p_{1},p_{2}\cdots p_{L}],p_{i}\in R^{N\times 1}$, where ***L*** is the channel number, ***K*** is the length of *x*. The feature *T* is obtained through an *S*×1 1D-LDSC layer with channel number *L*. After that, the characteristic information of local cross-channel activated graphs in *T* is acquired and projected through the ECA module to obtain the optimization vector ***V***. The feature $V=[v_{1},v_{2}\cdots v_{L}]$, where *v*_*1*_ belongs to the *i*th channel in the optimized vector *V* and takes on the value range is [0, 1]. The value of *v*_*i*_ denotes the significance of the *i*th channel. The enormous value of vi means that the channel is more correlated with the fault feature, and the smaller value means that the channel is less correlated with the fault feature. The fault features are enhanced through the ECA module, and the non-fault features are weakened to obtain the optimized features. Thus, the feature has better fault recognition.

### 2.3 Multi-scale branching attention feature extraction module (MBAFEM)

**Fig 5:**
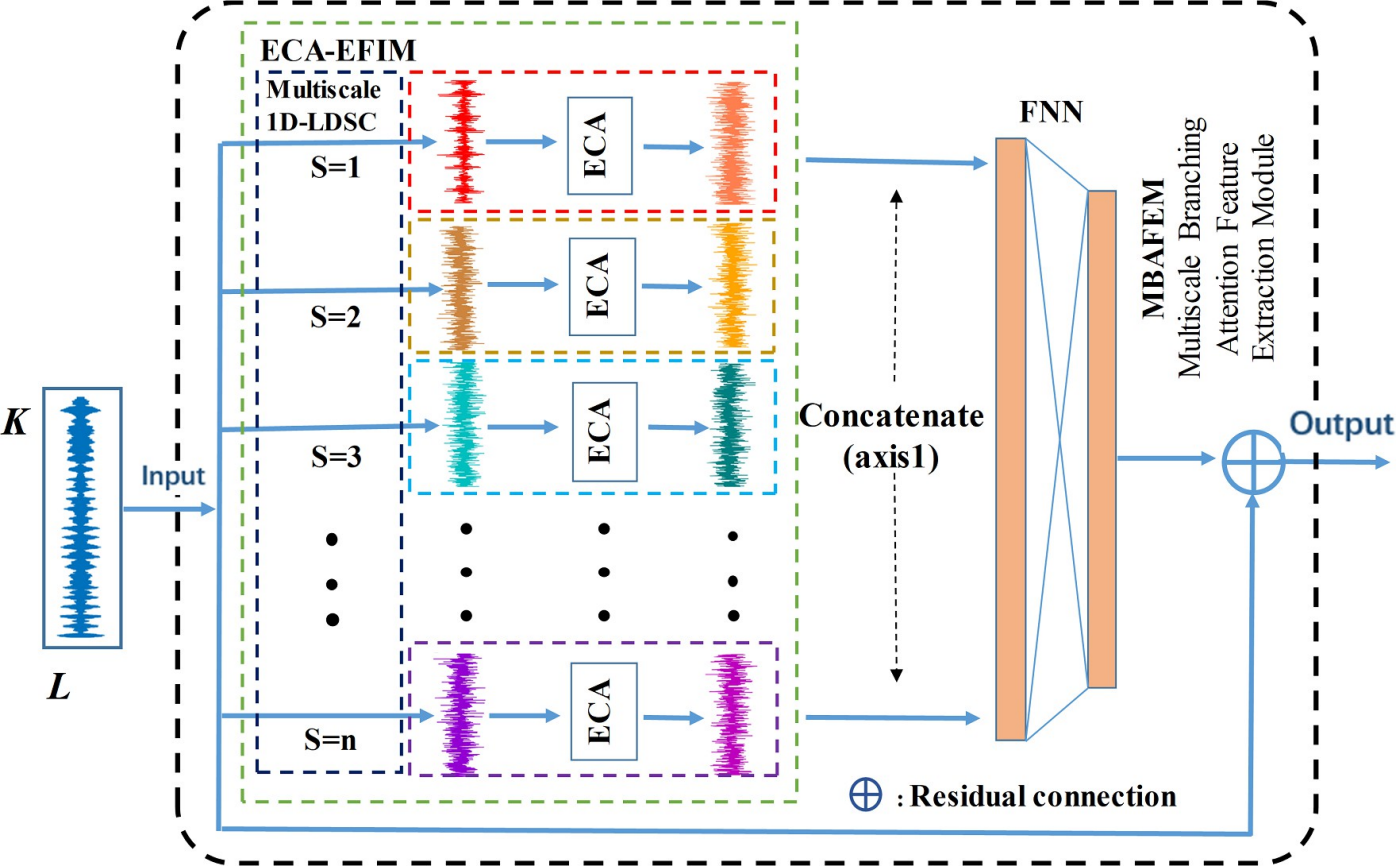
The structure of the MBAFEM.

As shown in Fig 5, We formed a multi-scale branching feature extractor by stacking multiple EFIM modules, whose primary purpose is to capture in parallel vibration signals complementary multiscale fault features by ECA-EFIM modules with different scale kernels (referred to as multi-scale 1D-LDSC in Fig 5). The initial extracted feature set {*T*_*1*_, *T*_*2*_,…, *T*_*N*_} is obtained by *N* parallel 1D-LDSC layers for various kernel sizes, where *T*_*j*_∈ℝT×(L/r)(*j = 1*,*2*,…, *N*) and *N* denotes the count of scales. In order to obtain detailed features for information extraction and to maintain the shape of *T*_*j*_ constant in the above operation, the step size of all 1D-LDCS layers is set to 1. To keep the *T*_*j*_ (*j = 1*, *2*,…, *N*) with the identical length, a padding strategy is used and the step length of all 1D-LDSC layers is set to 1. So that the *T*_*j*_ maintains its original shape in the above operation after the detailed feature information is extracted. The optimized feature set {*T*_*1*_^*’*^, *T*_*2*_^*’*^,…, *T*_*N*_^*’*^} is obtained by reconstructing the original feature set {*T*_*1*_, *T*_*2*_,…, *T*_*N*_} through the ECA-EFIM module, and the multi-scale feature map *T*_*m*_ contains all fault discriminatory information at different time scales and consists of *T*_*j*_^*’*^ concatenated along the channel dimension, which can be expressed as

$$T_{m}=[T_{1}^{\prime },T_{2}^{\prime },\cdots T_{N}^{\prime }]$$ (1)

Then, *T*_*m*_ is followed into a feedforward neural network with 2 fully connected layers of the network where the number of neurons is 256 and L, respectively, to extract valuable features further and remove redundant features. The third layer neuron is set to *F* to make the FNN output have the same size and shape as the input of EFIM. Finally, the residual connections are introduced to sum the FNN output and the EFIM input to obtain the final output of the module. The introduction of residual connections better reinforces primitive information. It prevents valuable information from being lost, in addition to overcoming the problems of gradient disappearance and performance degradation of deep networks.

### 2.4 Stacked MBAFEMS(S-MBAFEMS) based for fault diagnosis

It is observed from the literature [40] that deeper networks are good at extracting features with strong conditional expressiveness and robustness. To ensure better extraction of deeper and more abstract fault features from the noise environment, this paper constructs a deep Neural network of multiscale branching constructs a deep multiscale branching network (S-MBAFEMS) for rolling bearing fault diagnosis by superimposing multiple MBAFEMs.

**Fig 6:**
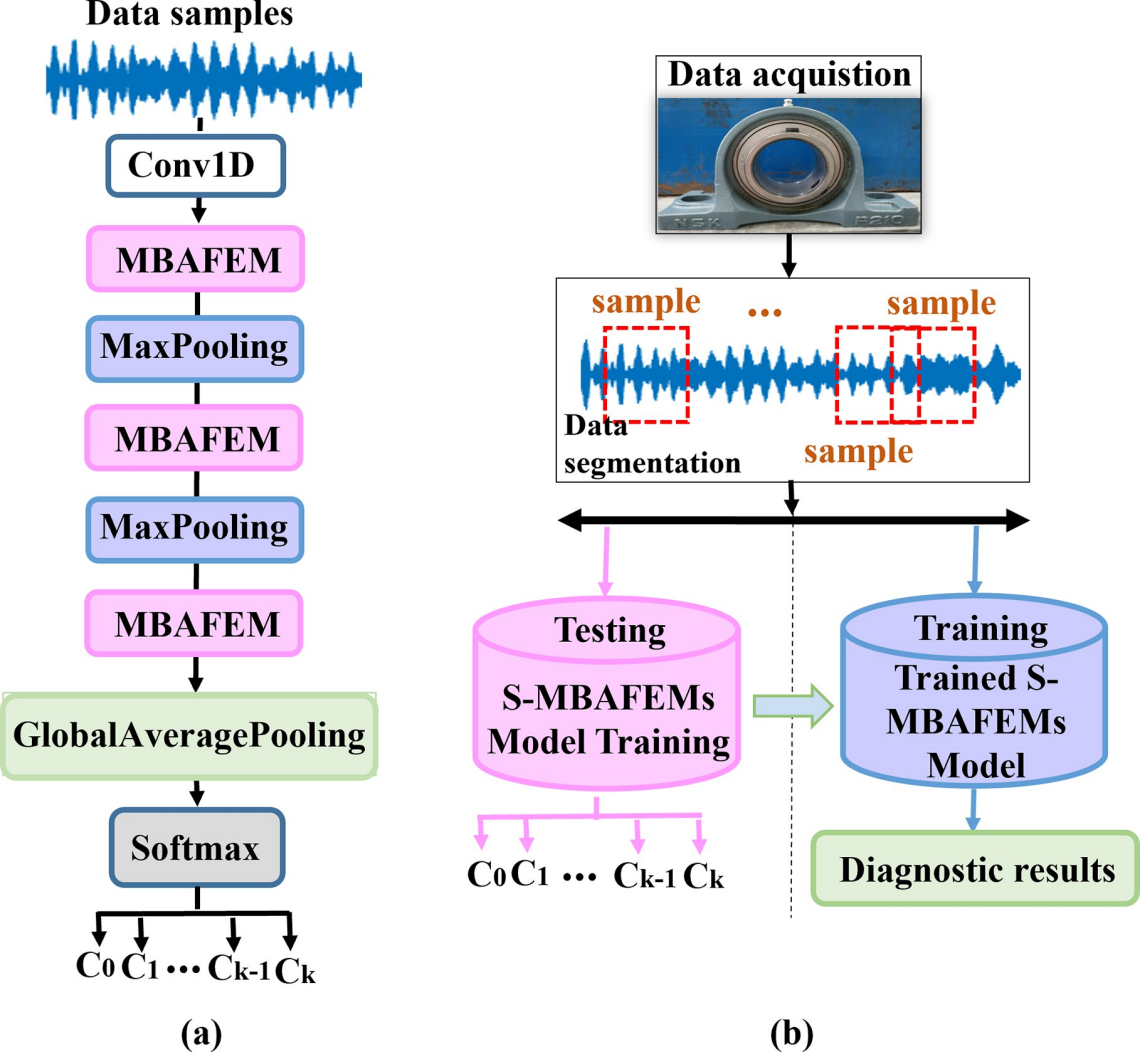
(a) Structure of the S-MBAFEMs, and (b) The overall flowchart of the S-MBAFEMs based fault diagnosis method.

The structure of S-MBAFEM is displayed in Fig 6(A), where three MBAFEMs are stacked. In this paper, we all directly use 1-dimensional time series of vibration data collected from the rolling bearing by sensors as the model’s input. First, various features of the one-dimensional vibrational signal are extracted by a one-dimensional convolutional layer (Conv1D) to extend its channel size. Further, Conv1D enables the remaining connection manipulation in the first MBAFEM of S-MBAFEMs. The kernel count of Conv1D is set to 64 in this paper, which can also be adjustable based on the actual situation. Then, the Conv1D output is delivered to the cascading MBAFEM. Between every two MBAFEMs, we employ a maximum pooling layer with pool lengths set to 2 to the maximum pooling layer to trap locally immutable features and speed up training. Between each of the two ELMFEMs, we use a maximum pooling layer with the pooling length set to 2 to trap locally constant features and expedite training. Finally, a typical fault classification module (GAP+softmax) is used as a fault diagnostician to output the classification results, the global average pool (GAP) layer is used to avoid overfitting, and the softmax layer is employed to export the critical probabilities of each category. Assume that the diagnostic network learns features of *Y* and has *k* classes of fault diagnosis tasks.


$$q_{j}=\frac{\exp(\theta^{j}GAP(Y))}{\sum_{j=1}^{k}\exp(\theta^{j}GAP(Y))},j=1,2,\ldots ,k$$ (2)

Where *θ*_*j*_ is the parameters of the full connection layer; *q*_*j*_ is the output probability of the class *j* and $\sum_{j=1}^{k}q_{j}=1$.

In this paper, some structural parameters in S-MBAFEM are set as follows: the kernel count of Conv1D is set to 64, the parameter v for dimensionality reduction was set to 8, and the quantities of neurons in the FNN layers was set to 256 and 64, respectively. Fig 6(B) shows the detailed procedures of the fault diagnosis method based on S-MBAFEMs. The specific steps of which are as follows:

Step 1: Acquisition of vibration signals of rolling bearings under multiple types of state characterization by acceleration sensors. After that, the vibration signal is split by multiple subsets of samples with the same sample length to generate dataset D for being trained.
Step 2: An end-to-end model for rolling bearing fault diagnosis is constructed by superimposing S-MBAFEMs modules. The original vibration signal is passed through Conv1D and is extracted as a multidimensional feature mapping fed into the stacked MBAFEM and classifier, which finally outputs the corresponding fault classification results. Offline training is completed when the number of training sessions reaches the maxi-mum number of epochs.
Step 3: To ensure the accuracy of the test experiment process, test samples are collected from the accelerometer of the rolling bearing using the same method as in step 1, which are fed into a well-performing online fault diagnosis model for diagnosis to obtain the current bearing status identification results.

## 3. Experimental validation setup and data description

### 3.1 CWRU dataset

**Fig 7:**
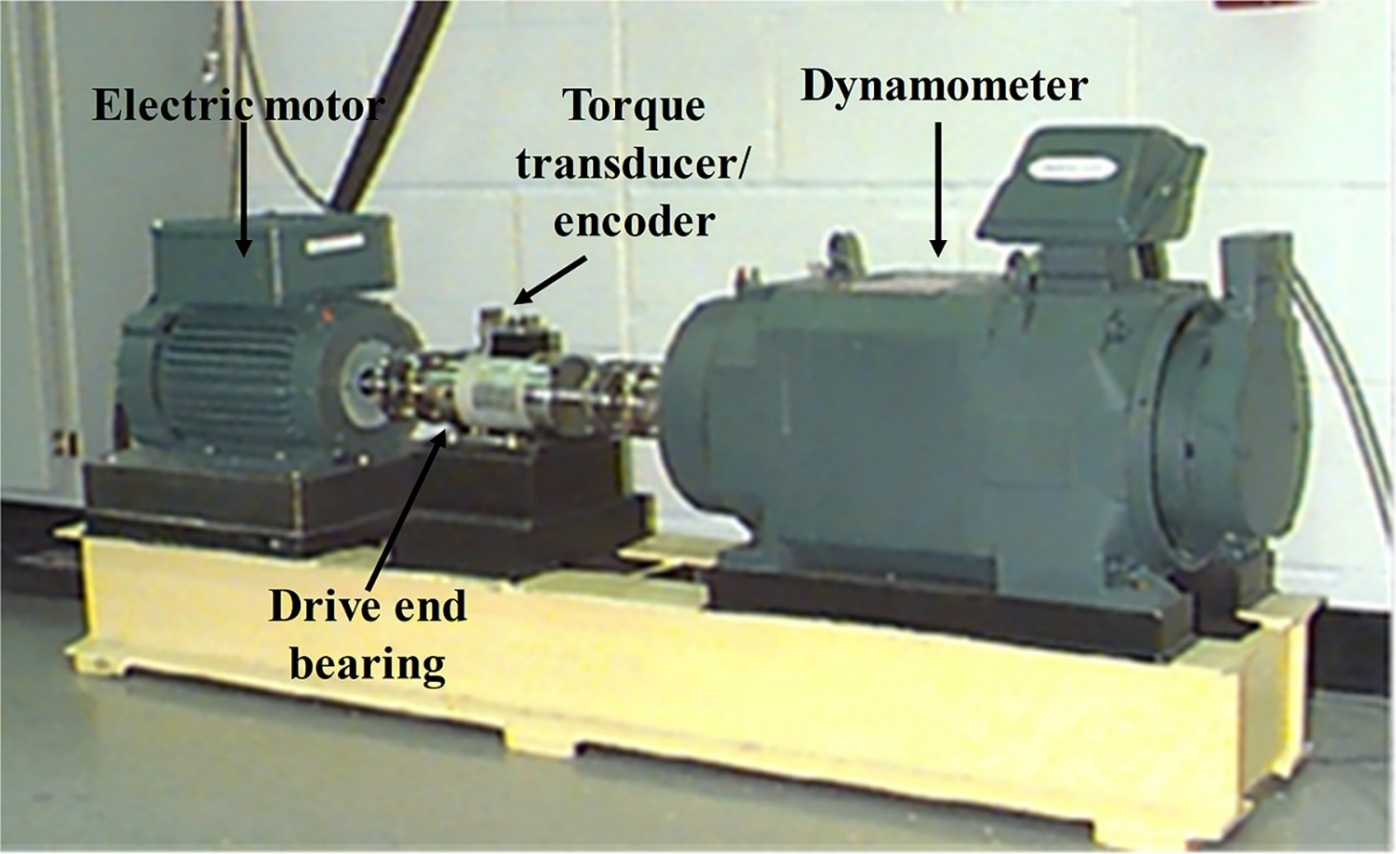
CWRU’s rolling bearing fault test bench.

The publicly available bearing dataset from CWRU [41] is used for experimental validation. The test bench is displayed in Fig 7. Vibration data were collected from the drive side at a sampling frequency of 12kHz. In addition to the normal condition, the data set contains three fault types: inner ring, outer ring, and ball. In addition, each fault category has three defect diameters and the bearing vibration signals are collected at each of the four speeds and loads.

Thus, the data set contains nine fault states and one normal state namely, normal condition (NC), inner ring failure (IF) (failure diameters: 7 mils, 14 mils, 21 mils), rolling element failure (RF) (diameter of failure: 7 mils, 14 mils, 21 mils) and outer ring failure (OF) (diameter of failure: 7 mils, 14 mils, 21 mils) for the rolling bearings are characterized. The vibration acceleration datasets used in this paper are list in Table 1.

**Table 1:** Description of the health conditions in the motor bearing dataset.

Label	Fault Location	Fault Size(/mil)	Load (/hp)
C0	NC	0	0,1,2,3
C1	IF	7	0,1,2,3
C2	IF	14	0,1,2,3
C3	IF	21	0,1,2,3
C4	OF	7	0,1,2,3
C5	OF	14	0,1,2,3
C6	OF	21	0,1,2,3
C7	RF	7	0,1,2,3
C8	RF	14	0,1,2,3
C9	RF	21	0,1,2,3

### 3.2 Paderborn University dataset

**Fig 8:**
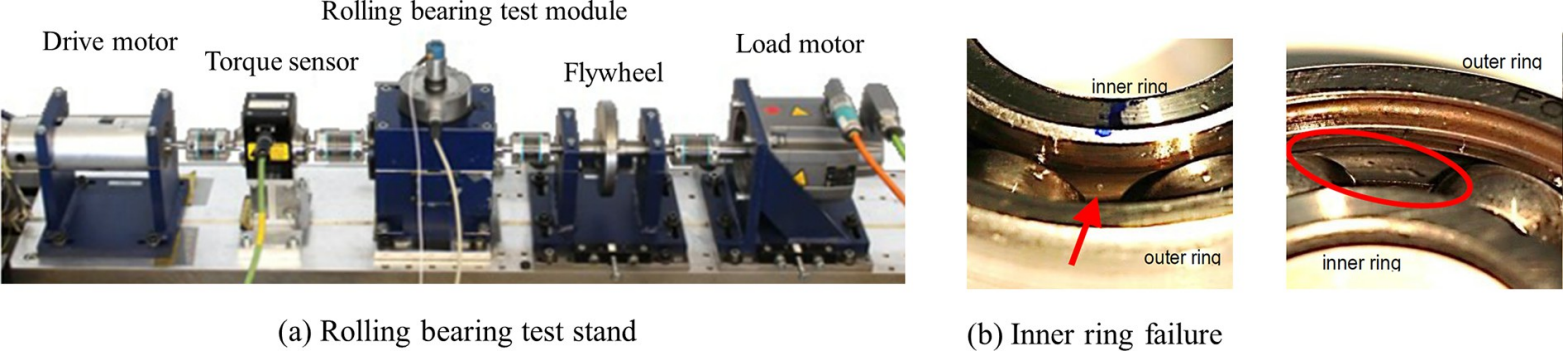
Test bench and faulty bearing in dataset 2.

Public dataset 2 was provided by KAT Data Center, University of Paderborn, Germany. The test bench used for this data acquisition is shown in Fig 8(A), with a sampling frequency of 64 kHz. The experimental bearing is a type 6203 deep groove ball bearing, and an accelerated life test obtains the damage path. The data used to build the diagnostic task contains seven health status categories: normal (N), three damage levels for inner loop failures (IF1/IF2/IF3), two damage levels for outer loop failures (OF1/OF2), and combined inner and outer loop failures (CF). The damage type of the faulty bearing is pitting corrosion, and the damage degree is divided according to the length of the damaged surface. Grades 1, 2, and 3 indicate damage lengths of 0~2mm, 2~4.5mm, and 4.5~13.5mm, respectively. The vibration dataset 2 used in this paper is shown in Table 2.

**Table 2:** Description of the health conditions in the rolling bearing dataset.

Label	Fault Location	Radial force (/N)	Load (/Nm)	Speed(/rpm)
C0	NC	1000	0.7	1500
C1	IF1	1000	0.7	900
C2	IF2	1000	0.7	900
C3	IF3	1000	0.7	900
C4	OF1	1000	0.1	1500
C5	OF2	1000	0.1	1500
C6	CF	400	0.7	1500

### 3.3 Experimental validation setup

Since the vibration data collected is a long time-series signal, we obtain more training samples by repeating the sliding coverage segmentation technique. With the above approach, 7500 samples of length 1200 were constructed based on the dataset, 80% of which were selected for model training and the rest were used for testing. To ensure the accuracy of the experiment, we repeat the experiment 10 times and statistical results.

F1 score and Accuracy are used as metrics to evaluate fault diagnosis performance. The accuracy is defined as Eq (3), and the F1 score is defined as Eq (4), respectively.


$$ACC=\frac{TP+TN}{TP+FN+FP+TN}$$ (3)

$$F_{1}=\frac{2\mathrm{TP}}{2\mathrm{TP}+\mathrm{FP}+\mathrm{FN}}$$ (4)

We incorporated Gaussian white noise in each sample to resemble the noise disturbance faced by the bearing in reality. The *SNR*_*dB*_ is defined in Eq (5).

$$\begin{array}{c} SNR_{\mathrm{dB}}=10\log_{10}(P_{\mathrm{signal}}/P_{\mathrm{noise}})\\ P_{\mathrm{signal}}=\frac{1}{N}\sum_{n=1}^{N}|v[n]{|}^{2} \end{array}$$ (5)

where *P*_*signal*_ and *P*_*noise*_ are the power of signal and noise, and *v[n]* is the noiseless signal, respectively. In reality, there is inherent environmental noise in the vibration signals received from the bearing bench, but we were unable to assess the intensity of the noise. To facilitate the experimental study, it is assumed in this paper that the vibration signals obtained directly from the test bench are noiseless, and the signals added to the noise at a later stage are noisy.

The study was implemented in the Pytorch and Python framework. A workstation with an Intel Core XS-4210 CPU, 16GB of RAM and an RTX 3090 GPU was used to run the code. The diagnostic model was computed using cross-entropy loss and was iterated 300 times by the Adam optimizer with a learning rate of 0.001 and a batch size of 128. In addition, a learning rate decay operation was used during the training process, i.e., the learning rate decreased at a rate of 5% every 25 epochs.

## 4. Results and discussion

In this section, the validity and superiority of the proposed S-MBAFEMS are verified and reviewed through multiple ablation experiments on the bearing dataset. First, we investigate the impacts of the size and number of S-MBAFEMs used on the diagnostic results to determine the optimal model structure for subsequent experiments. Second, we demonstrate the effectiveness of 1D dynamic convolution, 1D-LDSC, ECA-EFIM, and residual learning in improving model capabilities and suitability through comparative experiments. Finally, to fully evaluate the effectiveness and superiority of the proposed models, we selected several representative multiscale CNN frameworks in the field of intelligent fault diagnosis to perform the same diagnostic tasks.

### 4.1 Influence of the number of MBAFEMS and scale size on diagnosis performance

To verify the effect of the size and network depth of S-MBAFEM on the diagnostic capability, in this study, 10 different kernel designs were used to set the kernel size as 1×1, 4×1, 9×1, 16×1, 25×1, 36×1, 49×1, 64×1, 81×1, and 100×1 for all number of the Kernels of 16.

**Fig 9:**
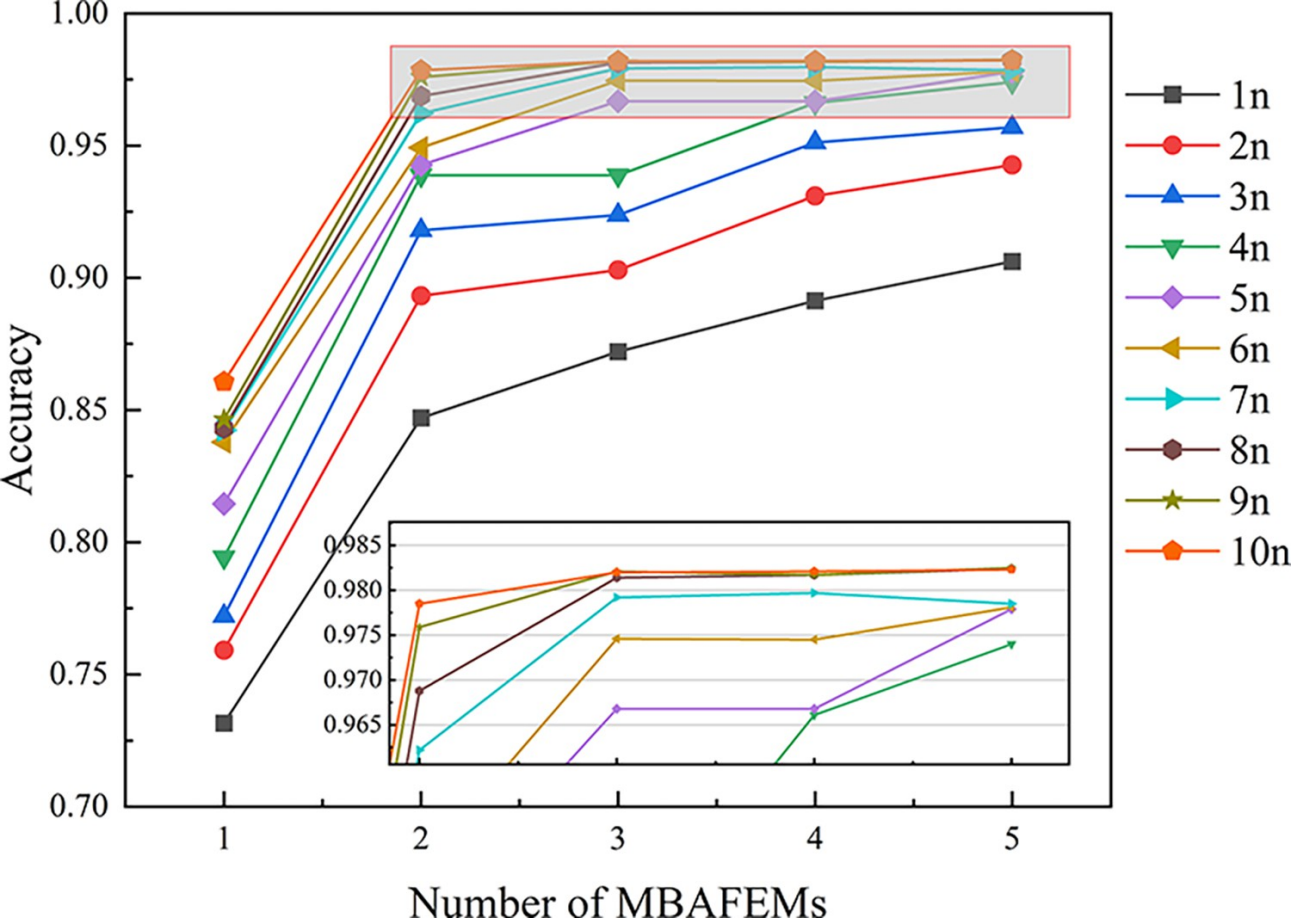
The effect of scale and the number of MBAFEMs on the performance using S-MBAFEMs.

In addition, we made different settings for the MBAFEMs are set differently, ranging from 1 to 5. The experiments were conducted at -6 dB of noise. The average results of the five experiments in each case are shown in Fig 9. In the results, 1n indicates that only 1×1 kernels are used, 2n indicates that 1×1 and 4×1 kernels are used, and as so forth, 10n the full 10 kernels are used. In general, the proposed model’s testing accuracy increases with the number of scale branches and MBAFEMs. The highest test accuracy of 98.25% was achieved when the fault diagnosis model consists of five MBAFEMs, each MBAFEM containing nine scales (indicated as model (9, 5)). This suggests that deeper and larger networks can acquire more holistic and depth features, resulting in the improved diagnostic performance of the model.

When the number of scales and MBAFEMs included in the model reaches a certain amount, the diagnostic test accuracy tends to be saturate if more scales and MBAFEMs are added. For example, the test accuracy of the model (8,3) improves by nearly 16.69% compared to model (5,1), however, the test accuracy of the model (10,5) improves by only about 0.06% compared to model (8,3).

The observation can be interpreted in two ways.1) Restricted by the number of training samples. When the amount of training samples is kept, and the model parameters are increased to a particular value, it is extremely difficult to continue optimizing these parameters. 2) Restricted by the training sample length. According to the structure of S-MBAFEM as the depth of the network increases, more maximum pooling layers are used, which significantly reduces the feature map length of the extracted features. Therefore, MBAFEM at the end of the model makes it difficult to extract useful features from the convolutional layers with wide kernels.

By fully considering the actual properties of the samples utilized in this paper, the diagnostic capabilities, and the model training speed, a model with a kernel number of 8 and an MBAFEM number of 3 is selected in this paper and is mainly used for subsequent experiments. In the unspecified case, references to MBAFEMs in this paper refer to the model (8,3).

In addition to quantitative evaluation of model diagnostic results, feature visualization is performed by the t-distributed stochastic neighbor embedding (t-SNE) technique to visualize the effect of scale number and network depth on model diagnostic performance.

**Fig 10:**
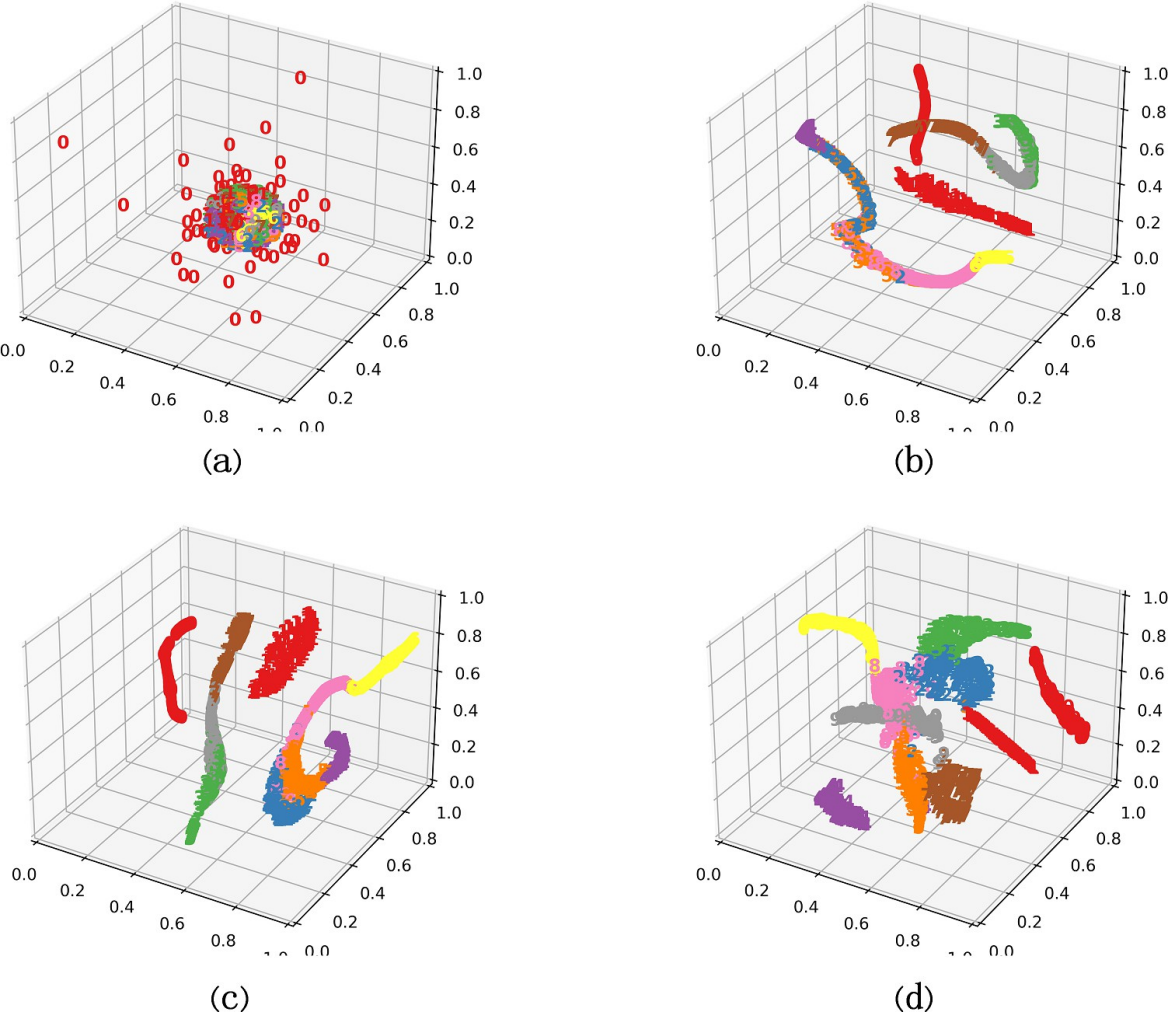
The proposed model is visualized with features under different health conditions: (a) input data, (b) model output consisting of a single scale (81 × 1) MBAFEM, (c) model (8, 1), and (d) model (8, 3).

As shown in Fig 10(A), all features overlap and are difficult to distinguish, which means that the feature information of the unprocessed input data is difficult to separate. In Fig 10(B), the features of the same health status are gradually clustered, and the characteristics of different health states are progressively divided. In Fig 10(D), it is clear that the various health conditions are clustered together, and only a few samples are misclassified.

In terms of the above, as the number of scales and MBAFEMs increases, the valuable features learned by the model and the features under identical conditions show better clustering performance. In general, the comparison of feature visualization validates the effectiveness and superiority of S-MBAFEMs to learn more discriminative fault features from the original vibration signals that are loaded with inherent environmental noise.

### 4.2 Effectiveness of lightweight dynamic separable convolution

**Fig 11:**
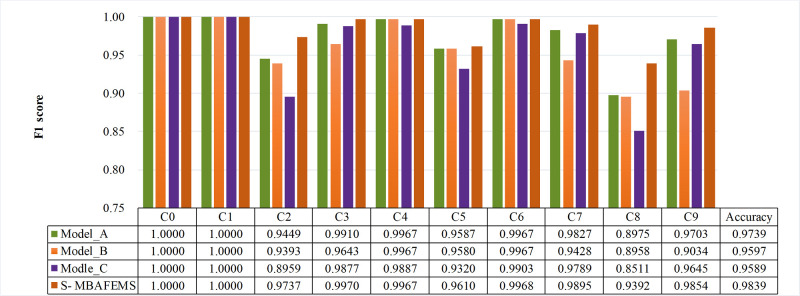
Methods compared results tested by the data with fault development.

In this paper, a LDSC method is proposed to increase in the expressiveness of the model while reducing the number of parameters. In this section, to verify the efficacy of the dynamically separable convolution, the standard convolution is used in the multi-scale branch extractor of Model-A concerning the proposed model. The model is tested using the same dataset and training strategy. Fig 11 shows the test results under different models, Clearly, the network model’s performance lacking the dynamically separable convolution degrades by 0.75% compared to the proposed model. The model is tested using the same dataset and training strategy. Fig 11 shows the test results under different models, Clearly, the network model’s performance lacking the dynamically separable convolution degrades by 0.75% compared to the proposed model. Tables 3 and 4 show the model test results with different numbers and scales of MBAFEM modules. Compared with standard convolution we have improved the general accuracy by 0–1.5% using lightweight dynamic separable convolution, and the model’s parameters can be reduced by up to 17%. The experimental results demonstrate that the proposed lightweight dynamic separable convolution can focus the fault feature mechanism well and extract the fault features in the initial dataset effectively without enhancing the model storage cost, which helps to improve the diagnostic performance of the model.

**Table 3:** Proposed model parameters and size for different number of scales. (The number of MBAFEMs is set to 3).

Number of ELMFEMs	Standard 1D convolution			1D-LDSC		
	Parameters	Size(MB)	Accuracy	Parameters	Size(MB)	Accuracy
1	59363	0.26	0.8413	68363	0.30	0.8721
3	94421	0.42	0.9115	112973	0.53	0.9238
5	177863	0.78	0.9501	184463	0.86	0.9668
7	337337	1.46	0.9702	298193	1.39	0.9792
9	600491	2.54	0.9746	469523	2.13	0.9821

**Table 4:** Proposed model parameters and size for different number of MBAFEMs. (The number of scales per MBAFEMs is set to 8).

Number of scales	Standard 1D convolution			1D-LDSC		
	Parameters	Size(MB)	Accuracy	Parameters	Size(MB)	Accuracy
1	151970	0.64	0.8331	125794	0.57	0.8431
2	303098	1.30	0.9567	250746	1.15	0.9688
3	454226	1.95	0.9683	375698	1.73	0.9814
4	605354	2.61	0.9776	500650	2.30	0.9817
5	756482	3.26	0.9790	625602	2.87	0.9824

### 4.3 Effectiveness of the ECA-EFIM

We validate the validity of ECA-EFIM through constructing two comparison models, namely S-MBAFEMs and Model-B. It is worth mentioning that Model-B only lacks ECA compared to S-MBAFEMs. Both models are trained and tested with the same data and using the same training strategy.

Fig 11 shows the average results of the five accuracies, showing the stability of the diagnostic performance. It is clear that compared with the model miss ECA module, the accuracy of our model introducing ECA is improved by 2.17%, and the experimental results prove that the ECA mechanism can extract the fault features in the initial dataset well, which helps to improve the model diagnostic performance.

### 4.4 Effectiveness of the residual learning

The performance of residual learning is evaluated by con-structing two comparison models, namely S-MBAFEMs and Model-C. It is worth mentioning that model-C lacks only residual knowledge compared to S-MBAFEMs. Both models are trained and tested using the same data and training strategy. Fig 11 shows the five accuracies’ average results, showing the diagnostic performance’s stability. The performance of S-MBAFEMs is always better than Model-C. Specifically, the accuracy is improved by 2.25% compared to Model-C.

**Fig 12:**
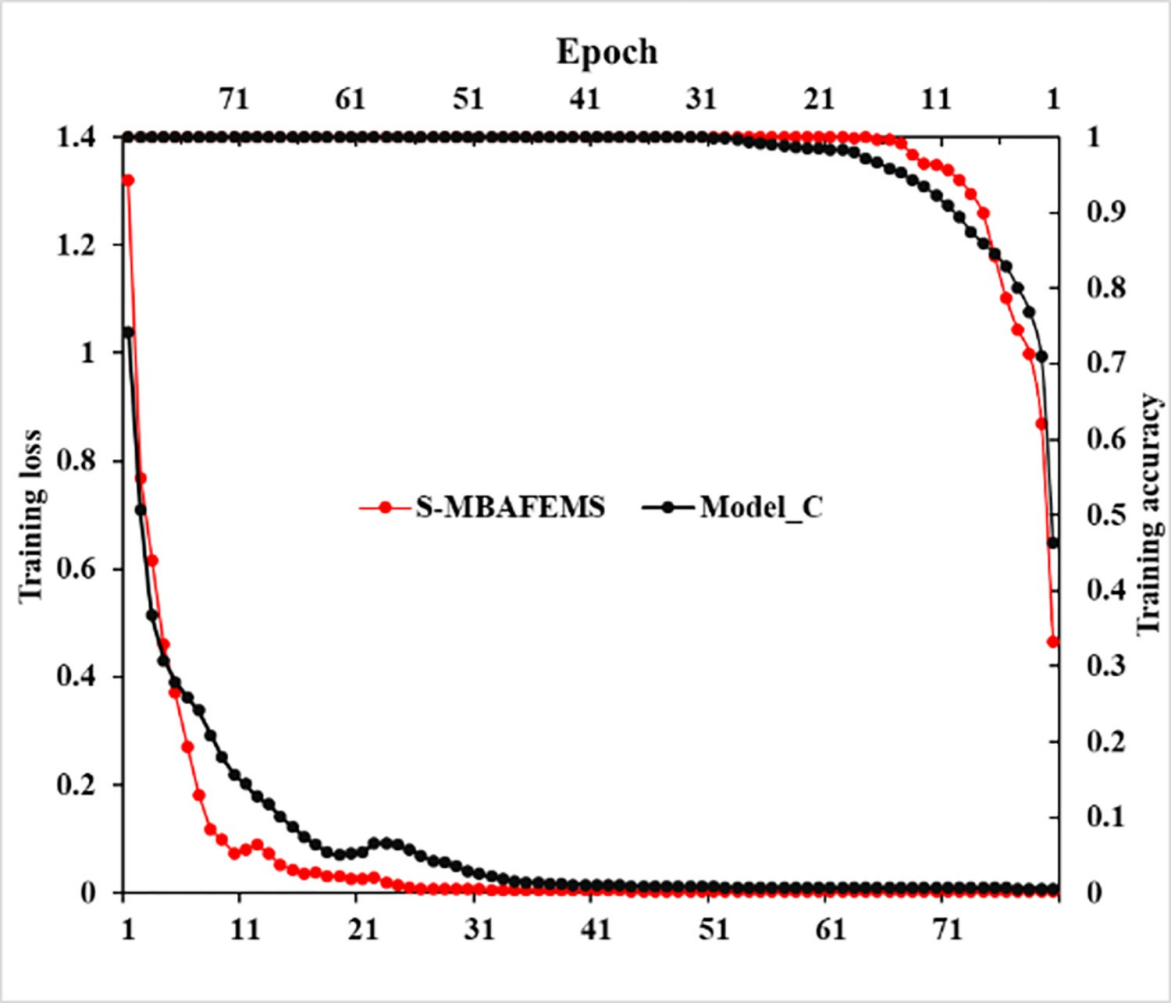
Training accuracy and training loss of different methods based on raw vibration signals.

The results show that the degradation of performance of S-MBAFEMs is alleviated effectively. Fig 12 illustrates the training accuracy and loss for the former 80 stages. It is observed that the test accuracy of S-MBAFEMs is slightly higher than that of Model-C, but both are almost 100%, both of which are higher than the test accuracy, which shows that S-MBAFEMs have better generalization ability. In addition, from the loss curve, compared with Model-C, the S-MBAFEMs are steeper, and the final value is smaller, which indicates that S-MBAFEMs can converge quickly in training. The result above further demonstrates that residual learning can effectively mitigate the effects of gradient explosion and performance degradation in deep networks and improve the effectiveness of model training.

### 4.5 Comparison with existing multiscale CNN models and existing machine learning

The following state-of-the-art approaches are implemented on the same diagnostic tasks for a comprehensive evaluation. MC-CNN [29] consists of one multiscale scale (100, 200, 300) information fusion layer and three convolutional pooling modules. MS-CNN [30] uses multiple coarse-grained layers to the original signal for processing. The features are then extracted using cascaded convolutional pooling pairs and a global average pooling layer. The IMS-FACNN [31] framework contains an input layer, an IMS coarse-grained program extraction layer, a feature recognition learning layer, and a classical classification layer.

**Fig 13:**
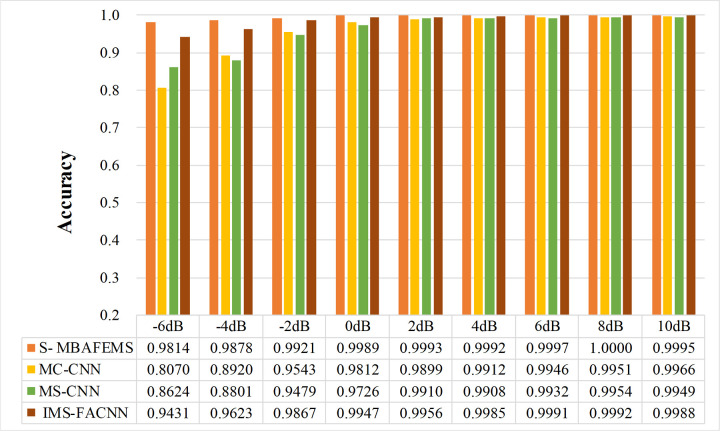
Performance of S-MBAFEMs and three comparison models in different noise environment.

The comparison experiments were conducted in various noisy environments with signal-to-noise ratios ranging from -6 dB to 10 dB. Each model had the same training strategy and used the same dataset. The results of the comparison experiments are presented in Fig 13. The diagnostic performance of S-MBAFEMs is better than the other three models. The diagnostic accuracy of present models exceeds 98% at the whole noise level(-6dB-10dB), and the accuracy of the model increases and stabilizes with the rise of SNR.

When the signal-to-noise ratio is 0db-10db, the accuracy of all models exceeds 97%. With the increase in signal-to-noise ratio, the model’s accuracy will increase and stabilize. At this time, the feature extraction ability of the model itself determines its diagnostic accuracy. As the signal-to-noise ratio decreases, the information in the signal is submerged by noise, and the fault diagnosis accuracy of the four models decreases. When the signal-to-noise ratio is -4dB, the fault diagnosis accuracy of MC-CNN and MS-CNN is lower than 90%. In contrast, the accuracy of the proposed model and the IMS-FACNN model is maintained above 96%. However, when the signal-to-noise ratio of the IAS model is -6dB, the ability of anti-noise interference decreases, and its accuracy is reduced by 1.92% compared with the SNR = -4dB.

And the accuracy of the proposed model at -6db is 98.14%, which is higher than the other models by 17.44%, 11.9%, and 3.83%, respectively. It can be seen that the proposed model has an excellent noise immunity performance.

### 4.6 Model generalizability analysis

**Fig 14:**
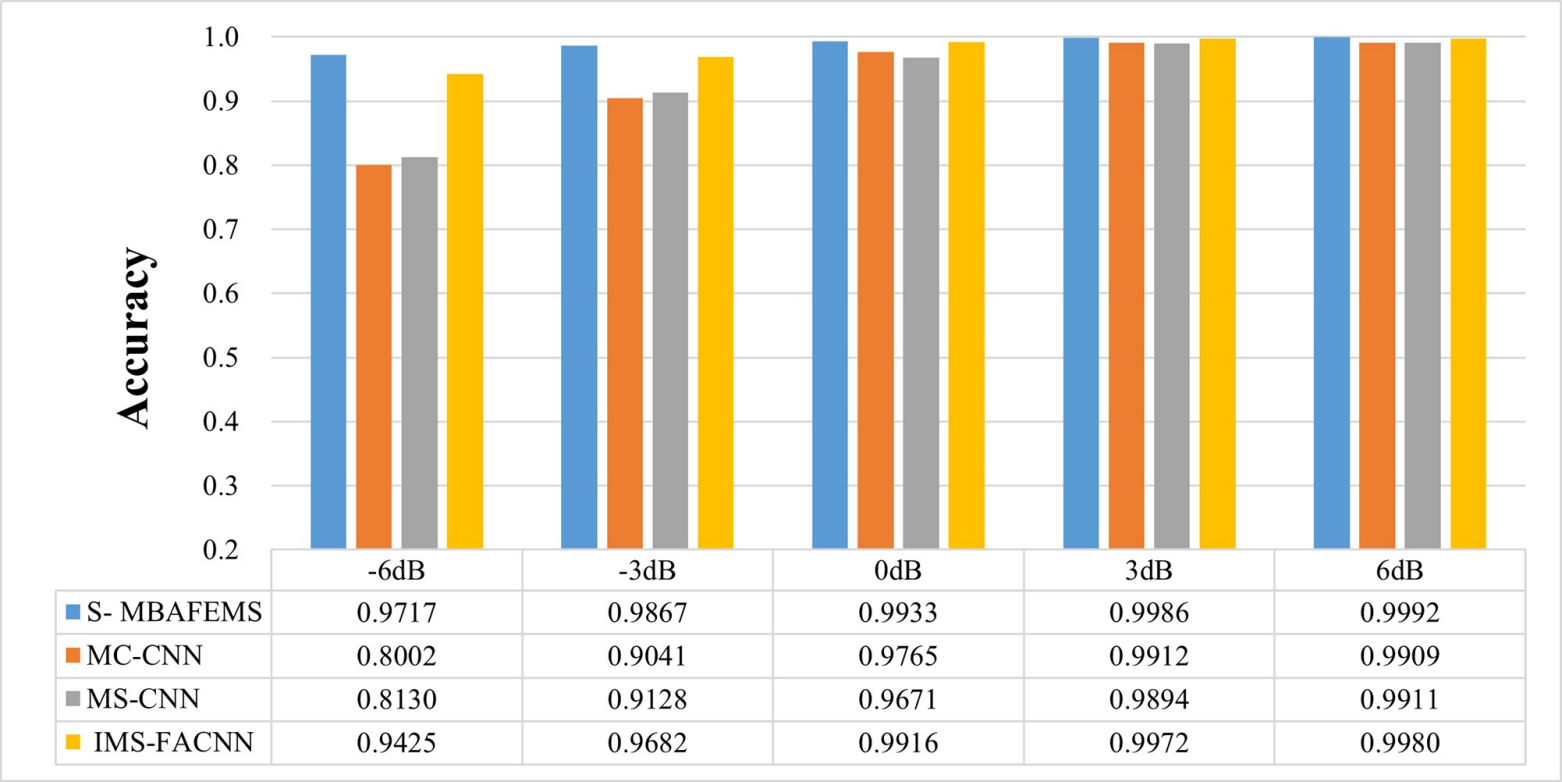
Model generalization performance analysis.

To verify the generalization performance of the proposed model, the rolling bearing data set of Paderborn University in Germany was used for experiments. The dataset is described in detail in subsection 2.2 of Section 2, with seven health states. The comparison model was consistent with the Case Western Reserve University dataset experiment. The comparison experiment was conducted in different noise environments, and the signal-to-noise ratio was -6dB ~ 6dB. Each model has the same training strategy and uses the same data set. The comparative test results are shown in Fig 14. In the data set of Paderborn University in Germany, the diagnostic performance of S-MBAFEMs is superior to the other three models. The model’s accuracy rate gradually increases and becomes stable with the increase in the signal-to-noise ratio. The diagnostic accuracy of the proposed model is over 98% at the whole noise level (-6dB-6dB), which is 0.83%-17.15%, 0.81% -15.87%, and 0.12%-2.92% higher than that of other comparison models, respectively. The model has good generalization performance.

### 4.7 Model computational complexity analysis

To verify the feasibility of the proposed model in practical industrial applications, the computational complexity of the proposed model was evaluated under the specified hardware and software conditions.

During the model’s training, the training speed is 3 min/batch due to the complex model parameters, large sample size, and long recursive training time. In contrast, during the testing of the model, the testing time is about 8.87ms/batch on average, and the computation time is less than 1 sec. The actual recognition speed of the trained model implanted in the specified computing environment meets the requirements of practical engineering applications.

## 5. Conclusions

An improved multi-scale branching convolutional neural network is proposed for rolling bearing fault diagnosis. The network appropriately combines 1D-LDSC, ECA-EFIM and residual learning to form complementary advantages. It reduces model storage and enhances generalization.

Experimental results based on CWRU dataset verify the effectiveness of the proposed method. In addition, we compared with the existing multi-scale CNN models and verified the model’s generalization performance by the Paderborn dataset. The results show that the proposed model achieved more than 97% accuracy with -6dB noise with significant advantages, which not only has a good ability of fault feature extraction and robust noise immunity but also has a lower computational cost and a relatively broad prospect for industrial applications.

In further work, we will explore the problem of adaptive parameter setting for multi-scale branching structures for different diagnostic tasks in a simulated dataset consistent with industrial reality in order to improve the generalization capability of the model.
